# Improving Thermal Regulation of Lithium-Ion Batteries by Poly(vinylidene fluoride-*co*-hexafluoropropylene) Composite Separator Membranes with Phase Change Materials

**DOI:** 10.1021/acsaem.4c03121

**Published:** 2025-01-22

**Authors:** João P. Serra, Guilherme Antunes, Arkaitz Fidalgo-Marijuan, Manuel Salado, Renato Gonçalves, Weidong He, Senentxu Lanceros-Mendez, Carlos M. Costa

**Affiliations:** † Physics Centre of Minho and Porto Universities (CF-UM-UP) and Laboratory of Physics for Materials and Emergent Technologies, LapMET, 56059 University of Minho, 4710-057 Braga, Portugal; ‡ Department of Organic and Inorganic Chemistry, 518636 University of the Basque Country UPV/EHU, 48940 Leioa, Spain; § Applications and Nanostructures, BCMaterials, Basque Center for Materials, UPV/EHU Science Park, 48940 Leioa, Spain; ∥ IKERBASQUE—Basque Foundation for Science, Bilbao 48009, Spain; ⊥ Centre of Chemistry, University of Minho, Braga 4710-057, Portugal; # National Key Laboratory of Science and Technology on Advanced Composites in Special Environments, and Center for Composite Materials and Structures, Harbin Institute of Technology, Harbin 150080, People’s Republic of China; 7 Institute of Science and Innovation for Bio-Sustainability (IB-S), University of Minho, 4710-053, Braga, Portugal

**Keywords:** separator membranes, PVDF-HFP, phase change materials, thermal regulation, lithium-ion batteries

## Abstract

Separator membranes with thermal regulation properties have been developed for battery systems by the addition of phase change material (PCM) microspheres within the polymer separator. Separator membranes based on PCM microspheres (acrylic core–shell particle) and polymer (poly­(vinylidene fluoride-*co*-hexafluoropropylene)), PVDF-HFP, composites were obtained by thermally induced phase separation (TIPS) with different amounts of PCM microspheres (4, 8, and 16 wt %). It is demonstrated that PCM content impacts the morphology of the separator membrane, leading to a decrease in the degree of porosity from 76 to 47%, the β-phase content from 86 to 78%, and the degree of crystallinity from 22 to 13%, with increasing PCM content, leading to variations in electrolyte uptake and electrochemical characteristics of the membranes. Li/C-LiFePO_4_ half-cells were produced, and the best cycling behavior was achieved for the membrane with 16 wt % of PCM microspheres, showing 87 mAh.g^–1^ after 200 cycles and 2C-rate without capacity fade. Consequently, this work demonstrates a separator membrane with PCM materials with low thermal shrinkage and consequently robust mechanical characteristics, showing thermal regulation properties that can be used in the next generation of safer lithium-ion batteries.

## Graphical abstract

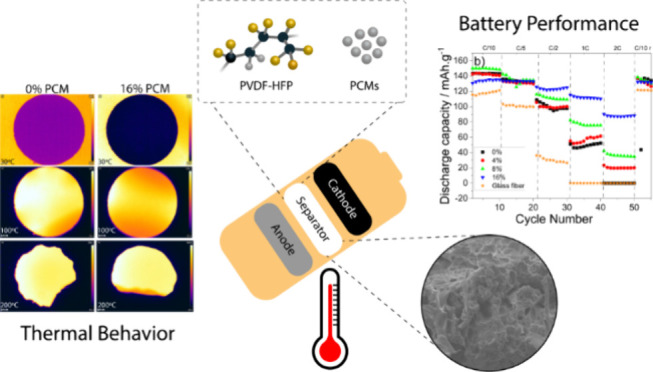

## 1 Introduction

The world is rapidly changing based on the strong technological development experienced throughout the 21st century.^1^ This accelerated technological development has increased people’s quality of life and occurs in a context of population growth,^2^ leading to large pressure on natural resources and increased pollution of our planet Earth.^3^

In the coming years, a range of critical challenges facing Earth will need to be addressed. To tackle these, the United Nations (UN) introduced in 2015 a comprehensive set of goals and targets, aiming to create a sustainable future by 2030.^4^ One of the goals of this 2030 Agenda is ensuring access to clean and affordable energy, promoting the energy transition in order to boost sustainable development in other areas such as agriculture, transport, education, and business, among others.^5^

The energy transition involves discouraging the use of fossil fuels (coal, natural gas, and oil) as the main source of energy production, fostering the use of more sustainable forms of energy production such as renewable energies, including solar, wind, hydroelectric, and nuclear power.^6^

This transition is a lengthy process since it involves various aspects such as the economy, politics, culture, demographics, and social development, among others.^7^ Several renewable energy sources, such as sun, wind, and water, are dependent on favorable environmental factors to maintain constant energy levels, which is one of the main limitations of these energy sources.^8^ An alternative to this limitation is to integrate energy storage systems into these energy production systems so that excess of produced energy can be stored to be supplied in the event of power failure or in peaks of higher energy demand.^9^

There are various energy storage systems, including electrochemical systems such as flow batteries, lead acid batteries, zinc batteries, and lithium batteries, among others.^10^ Of all tof hese types of batteries, lithium-ion batteries (LIBs) have come to the fore in recent years due to their high energy and power densities, extended lifecycles, efficiency, low self-discharge rate, durability, and robustness.^11^ Thus, they are widely used in different applications such as smartwatches, smartphones, computers, and electric vehicles.^12^

LIBs consist of two electrodes, a cathode and an anode, which are located on different metal current collectors (usually copper and aluminum) with a separator, which is typically a porous polymeric material soaked in an electrolyte solution,^13^ between them. This system is a closed and isolated system due to the instability of lithium in contact with oxygen from air and water and is housed in an impact- and deformation-resistant nickel-coated steel casing.^14,15^

Although LIBs are closed and isolated systems, there have been several accidents involving this energy storage system in recent years.^16^ These accidents result from manufacturing defects, accumulation of gases from constant oxidation–reduction reactions in the electrodes, short circuits due to defects in the separators, electrical or mechanical abuse, cooling, heat/gas generation, thermal shock, and thermal runaway, leading to damage to the structure of the electrochemical cell which can result in smoke, fire, or even explosion.^17^

The prevention of these accidents involves developing prevention mechanisms for both the individual cells and/or for systems made up of a set of cells.^18^ Mechanisms such as the insertion of thermal barrier layers between cells to prevent thermal runaway have already been studied, improving the thermal stability of the materials that make up the battery components.^19^ Surface coating of the materials to improve the interfaces or doping with heat dissipating materials or fillers that create films and block electrochemical reactions have been implemented, as well as physical protection mechanisms with safety valves and current fuses, among others.^20^

One of the materials that can provide a solution to thermal related battery safety problems, including overheating and thermal runaway, are phase change materials (PCMs). PCMs are materials that can change their state from solid to liquid and vice versa, releasing and storing thermal energy, and are considered one of the most effective for a wide range of applications due to their low cost, simple structure, and high cooling efficiency.^21,22^ These materials can be applied to LIB components and thus prevent thermal runaway.

This work describes the use of PCMs to generate passive thermal management through their incorporation into porous polymeric material used as a separator in LIBs. This will enable heat regulation and will allow improvement of battery safety. This specific PCM was chosen because of its melting temperature at 29 °C. Further, due to its low crystallinity value, poly­(vinylidene fluoride-*co*-hexafluoropropylene) is one of the most often used polymer matrices in the field of battery systems. The incorporation of these particles is used to improve battery safety without affecting the performance of this energy storage system, since the PCM captures and stores energy initially in the form of perceptible heat and then in latent form after reaching the phase transition temperature. After the transition temperature is reached, stored energy is released as the temperature drops. Different characterization techniques were used to evaluate the morphology, chemical structure, thermal behavior, mechanical properties, and electrochemical characteristics of the porous membranes with different PCM concentrations, and the results were correlated to battery performance variations.

## 2 Experimental Section

### 2.1 Materials

Poly­(vinylidene fluoride-*co*-hexafluoropropylene) (Kynarflex PVDF-HFP 2801-00107) was obtained from Arkema. Microencapsulated phase change materials (PCMs; acrylic core–shell particles, CrodaTherm ME 29P) with a melting temperature at 29 °C were obtained from Croda International PLC, U.K. The solvent *N*,*N*-dimethylformamide (DMF, 99.99%) was acquired by Fisher Chemical. *N*-Methyl-2-pyrrolidone (NMP, 99%), electrolyte solution of 1 M LiPF_6_ in ethylene carbonate–dimethyl carbonate (EC-DMC, 1:1 (vol)), and lithium metallic were purchased from Sigma-Aldrich.

The carbon black (Super P-C45), C-LiFePO_4_ (LFP), and poly­(vinylidene fluoride), Kynar PVDF HSV900, were acquired from Timcal Graphite & Carbon, Phostech Lithium and Arkema, respectively, for cathode electrode preparation.

### 2.2 Separator Membranes Preparation

PVDF-HFP composite membranes were developed by varying the weight percentage of microencapsulated PCM (4, 8, and 16 wt %). Figure 1 schematically represents the experimental process.

**Figure 1:**
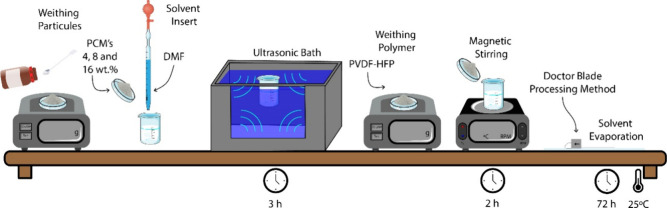
Schematic representation of the composite membranes’ preparation procedure.

The PCMs were first dispersed in DMF solvent (6 mL) for 3 h, and after complete dispersion, 1 g of PVDF-HFP was added to the solution. Each solution was magnetically stirred for 2 h at room temperature (25 °C) until the polymer was completely dissolved. The solution was then spread on a glass substrate by using the doctor blade method. After spreading, the membranes were solvent evaporated at room temperature (25 °C) for 3 days, in order to obtain a porous morphology by complete solvent evaporation.^23^ The thickness of the obtained membranes is in the range 50–80 μm. Membranes are designated by the microencapsulated PCM content.

### 2.3 Membranes Characterization

For the morphological evaluations, the membranes are previously coated with a thin gold layer by magnetron sputtering (Polaron, E 6700 model). Scanning electron microscopy was carried out with a NanoSEM-FEI Nova 200 system with an accelerating voltage of 10 kV.

Using a Quantachrome Instruments Poremaster-60 GT setup, mercury intrusion porosimetry (MIP) was carried out in the pressure range from vacuum (10^–4^ MPa) to 414 MPa.

The vibrational spectra of the membranes were investigated by Fourier transformed infrared spectroscopy in attenuated total reflection mode (FTIR-ATR) (PerkinElmer SpectrumTwo equipment) by applying 16 scans in the 400–4000 cm^–1^ range at a resolution of 4 cm^–1^. Equation 1 was applied for the calculation of the relative fraction of the polymer’s polar β-phase:^24^

1 $$F\left(\beta \right)=\frac{A_{\beta }}{1.26A_{\alpha }+A_{\beta }}$$

where *A*_*α*_ and *A*_*β*_ are the absorption intensities at 766 and 840 cm^–1^, respectively, corresponding to vibrations associated with the α and β crystalline phases of the polymer.

Using Netzsch STA 449 F3 Jupiter equipment, differential scanning calorimetry (DSC) was performed in a nitrogen atmosphere at 10 °C·min^–1^ in the 25–200 °C temperature range.

Using eq 2, the degree of crystallinity (*X*_c_) of the semicrystalline polymer was determined:^25^

$$X_{c}=\frac{\Delta H}{\Delta H_{\alpha }x+\Delta H_{\beta }y}$$ 2

where Δ*H* is the melting enthalpy of the membrane, Δ*H*_*α*_ (93.07 J·g^–1^) and Δ*H*_*β*_ (103.4 J·g^–1^)^26^ are the melting enthalpies of the α and β phases and *x* and *y* correspond to the amount of these phases present in the membrane, respectively, calculated after the FTIR results (eq 1).

Thermal imaging analyses were carried out for all membranes. The membranes were sliced into 19 mm diameter discs, and 5 cm × 5 cm copper foils were used as substrates. Heat moved through the copper foil and through the separator as the temperature increased. Using forward-looking infrared (FLIR) (A600-Series, Sweden), infrared light in the 7.5–13 μm band could be identified to obtain images at 7.5 Hz.

The imaging was conducted by using a noise-equivalent temperature difference mode with a 17 μm lens. Dispersion and heat transfer images were obtained in the separators at different temperatures.

### 2.4 Electrochemical Properties

Equation 3 was used to determine the uptake (ε) value as a function of time after the membranes were submerged in a 1 M LiPF_6_ in EC:DMC solution:

3 $$\varepsilon =\left(\frac{M-M_{0}}{M_{0}}\right)\times 100$$

Here, *M*_0_ and *M* are the membrane weights before and after being submerged in the electrolyte solution, respectively.

The ionic conductivity (σ_i_) of the porous membranes was measured by using a Swagelok cell with a Palmsens 4 potentiostat system in a sandwich configuration with the separator membrane placed between two stainless steel electrodes.

Impedance measurements were performed in the frequency range from 65 kHz to 500 mHz, and eq 4 was applied to obtain the ionic conductivity of the membranes:

4 $$\sigma_{i}=\frac{t}{AR_{b}}$$

where *t* and *A* represent the membrane’s thickness and area and *R*_b_ is the bulk resistance found by the intercept of the slanted line of the real impedance (*Z*′) with the imaginary impedance (minimum value of *Z*″).

Tortuosity (τ) and MacMullin number (*N*_M_) were calculated by eqs 5 and 6, respectively:

5 $$\tau =\sqrt{\frac{\sigma_{0}\varepsilon }{\sigma_{i}}}$$

6 $$N_{M}=\frac{\sigma_{0}}{\sigma_{i}}$$

where σ_0_ is the ionic conductivity of the 1 M LiPF_6_ in EC:DMC solution (11.6 mS·cm^–1^ at 25 °C), σ_i_ denotes the membrane’s ionic conductivity after the uptake process, and porosity is represented by ε.

The Li-ion transference number (*t*_Li^+^_) was measured at room temperature using symmetric cells,^27^ sandwiching a membrane (10 mm diameter) between two Li metal electrodes (8 mm diameter). For this experiment, a potentiostat/galvanostat Biologic VMP3 setup was employed, and *t*_Li_^+^ was calculated using eq 7 and the Bruce and Evans technique:^28,29^

7 $$t_{{\mathrm{Li}}^{+}}=\frac{I^{s}\left[\Delta V-I^{0}R^{0}\right]}{I^{0}\left[\Delta V-I^{s}R^{s}\right]}$$

where *I* ^0^ and *I* ^s^ are the initial and steady currents, respectively, and *R* ^0^ and *R* ^s^ are the corresponding resistance values of the Li electrode/electrolyte interfacial layers.

### 2.5 Battery Assembly and Evaluation

Using the synthesized porous membranes as separators (10 mm in diameter) submerged in an electrolyte solution inside an argon-filled glovebox, Li/C-LiFePO_4_ half-cells of the Swagelok type were developed. The cathode electrode was C-LiFePO_4_, and the anode electrode was metallic lithium foil, both with a diameter of 8 mm. NMP solvent was combined with 80 wt % C-LiFePO_4_, 10 wt % carbon black, and 10 wt % PVDF to fabricate the cathode. The electrode slurry was cast onto aluminum foil by doctor blade and left to dry for 2 h at 80 °C. The cathode active mass varies between 3.5 and 4 mg·cm^–2^.^30^

Using Landt CT2001A equipment, charge–discharge tests were performed on all samples at room temperature in the voltage range from 2.5 to 4.2 V, with current rates ranging from C/8 to 2C (C = 170 mA.g^–1^). For each membrane, a minimum of 6 batteries were tested. Electrochemical impedance spectroscopy (EIS) was performed with a Palmsens 4 potentiostat apparatus to ascertain the electrical characteristics of the Li/C-LiFePO_4_ half-cells containing the developed membranes. The EIS was carried out with a voltage signal amplitude of 10 mV AC in the frequency range from 10 mHz to 1 MHz.

## 3 Results and Discussion

### 3.1 Membranes Morphology and Degree of Porosity

The morphology of the neat and composite membranes with different contents of microencapsulated PCM obtained by TIPS is shown in the cross-sectional and surface SEM images in Figure 2. It is observed that all membranes present a porous morphology with interconnected pores typical of the PVDF-HFP polymer processed under the selected conditions.^25^ The main reason for this morphology is explained by the phase diagram of the binary polymer–solvent system, where liquid–liquid phase separation^25^ is obtained when solvent evaporation occurs in a polymer/solvent ratio (15/85) solution. Further, at the selected temperature of 25 °C, the polymer chains have reduced mobility that prevents the polymer from occupying the free space left by the solvent.^25^

**Figure 2:**
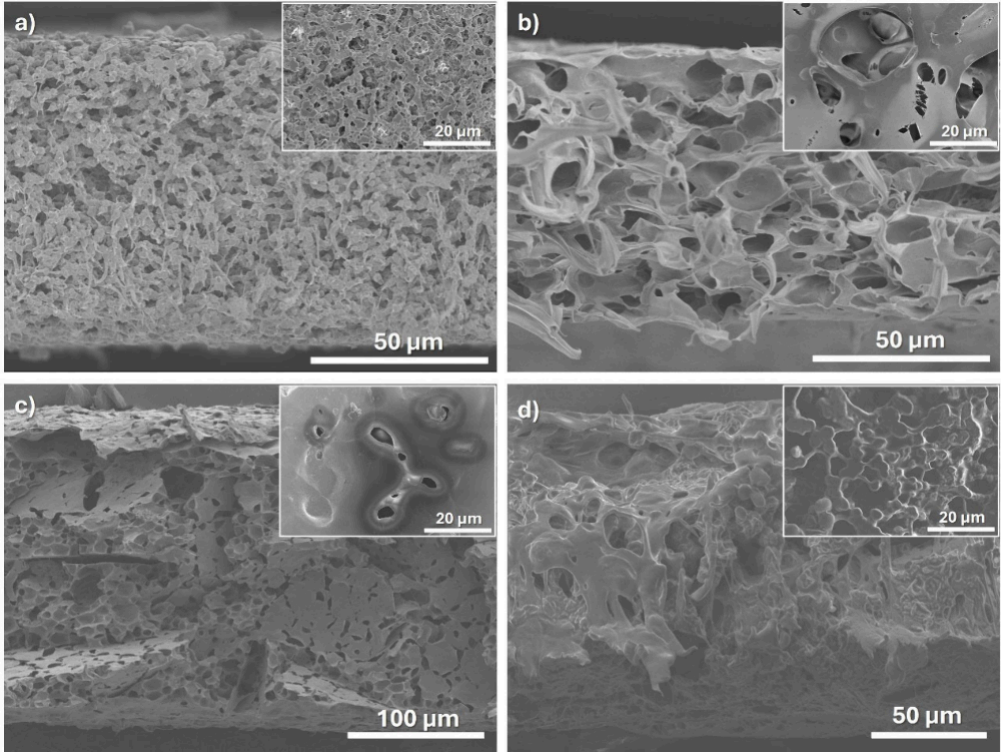
SEM images of the cross-section and surface (inset) of the different PVDF-HFP composite membranes with (a) 0, (b) 4, (c) 8, and (d) 16 wt % microencapsulated PCM content.

It is also observed that the inclusion of microencapsulated PCM affects the morphology of the membrane (Figure 2b–d), i.e., the size of the pores and their distribution, along both the cross-section and the surface of the samples. Figure 2 shows that, in general, the pore size and porosity decrease with the addition of microencapsulated PCM content, except for the membrane with 4 wt % PCM. PCM particles can aid in the phase separation process at lower filler concentrations due to their interactions with the polymer matrix, which result in larger pore development.^31^

The presence of the PCM particles hinders both the phase separation and the crystallization process (reduction of the porosity, Figure 3, and degree of crystallinity, Table 1), the fillers acting as defective structures during both processes.^32^

**Figure 3:**
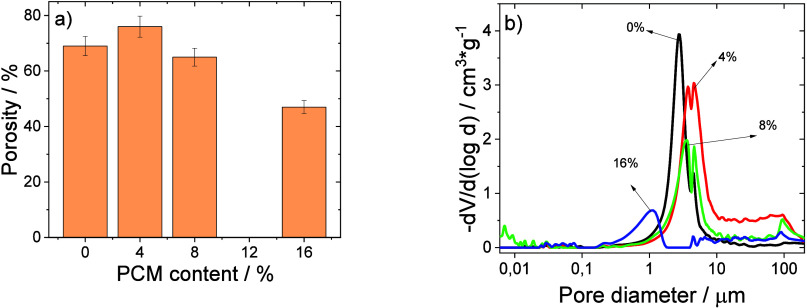
(a) Porosity and (b) intruded cumulative volume as a function of pore diameter for the different percentages of PCM on PVDF-HFP composite membranes.

**Table 1:** β-Phase and Degree of Crystallinity of the Developed Membranes

sample/(wt %)	β-phase/±2%	χ_c_/±1%
0	81	22
4	78	24
8	86	14
16	83	13

This behavior is demonstrated through the porosity values present in Figure 3a, obtained by Hg porosimetry, which ranges from 69 to 47% for the pristine membrane and the membrane with 16 wt % PCM, respectively

The logarithm of the cumulative intruded volume as a function of the pore size diameter for all membranes is shown in Figure 3b.

In Figure 3b, it can be observed that neat PVDF-HFP and PVDF-HFP composite membranes with 4 and 8 wt % PCM show a pore diameter distribution between 1 and 10 μm, but with a different relative size distribution. The PVDF-HFP membrane with 16 wt % PCM shows a distribution size range below 1.5 μm and down to 0.3 μm, with a lower pore content.

Overall, the obtained morphology and porosity of all membranes are suitable for lithium-ion battery separators.

### 3.2 Infrared Spectra and Thermal Analysis

The infrared spectra of the membranes are shown in Figure 4a, where the vibration peaks of the crystalline phases, α-phase at 766 cm^–1^ and β-phase at 840 cm^–1^, are observed and identified. Figure 4a also shows that the presence and content of the PCM do not affect the vibration peaks of the α-phase and β-phases of the polymer matrix. The β-phase content was obtained from eq 1 and is presented in Table 1. In all cases, the β-phase content is above 78%, indicating highly polar and piezoelectric membranes, independent of the PCM content. The crystallization of the polymer in the electroactive phase is determined therefore by the solvent evaporation temperature and the polymer/solvent ratio,^25^ with no effect by the presence of the PCM.

**Figure 4:**
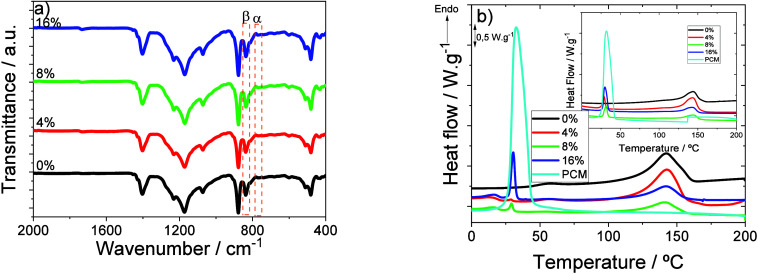
(a) FTIR spectra and (b) first scan of the DSC thermograms (inset second scan) for the prepared membranes.

Figure 4b shows the DSC thermograms of the different composite membranes, including the data corresponding to pristine PCM. In the case of neat PVDF membranes, a single peak is observed at 144 °C corresponding to the melting transition of the semicrystalline polymer. In the composite membranes, on the other hand, two melting events observed, at ∼28.8 and 144 °C, correspond to the melting of PCM microspheres and polymer, respectively.^25,33^ It should be noticed that the melting temperatures of both PCM and polymer are unaltered in the composites, showing the lack of strong physical or chemical interactions leading to modification in the thermal behavior. The melting behavior of the PCM and polymer is also detected in the second scan (inset of Figure 4b), demonstrating the reversibility of both processes. From the melting enthalpy of the polymer, the degree of crystallinity was calculated by eq 2, and the results are shown in Table 1. The degree of crystallinity decreases from 24% for the pristine polymer to 13% for the sample with the highest PCM content, the PCM acting as physical defects during polymer crystallization. It should be noticed that a decreases in crystallinity is typically beneficial for battery applications at it used to be correlated with increasing ionic conductivity values.^34^

According to the TGA curves (Figure S1 in the Supporting Information), both membranes exhibit stability up to 200 °C. The mass loss stage between 400 and 500 °C is observed in all membranes, and it is related to the degradation of the polymer, related to the chain-stripping of PVDF-HFP’s carbon–hydrogen (CH) bond scission.^35^ Further, the composite membranes present also a mass loss at 210 °C, caused by the breakdown of the paraffin wax in the PCM core.^36^

Figure 5 shows the optical images obtained from forward looking infrared radiometry (FLIR) to determine the temperature distribution of the separators at different temperatures. For the composite separator membranes with 8 wt % microencapsulated PCM content, the optical images are not shown as the behavior is similar to that observed for separator membranes with 16 wt % PCM content.

**Figure 5:**
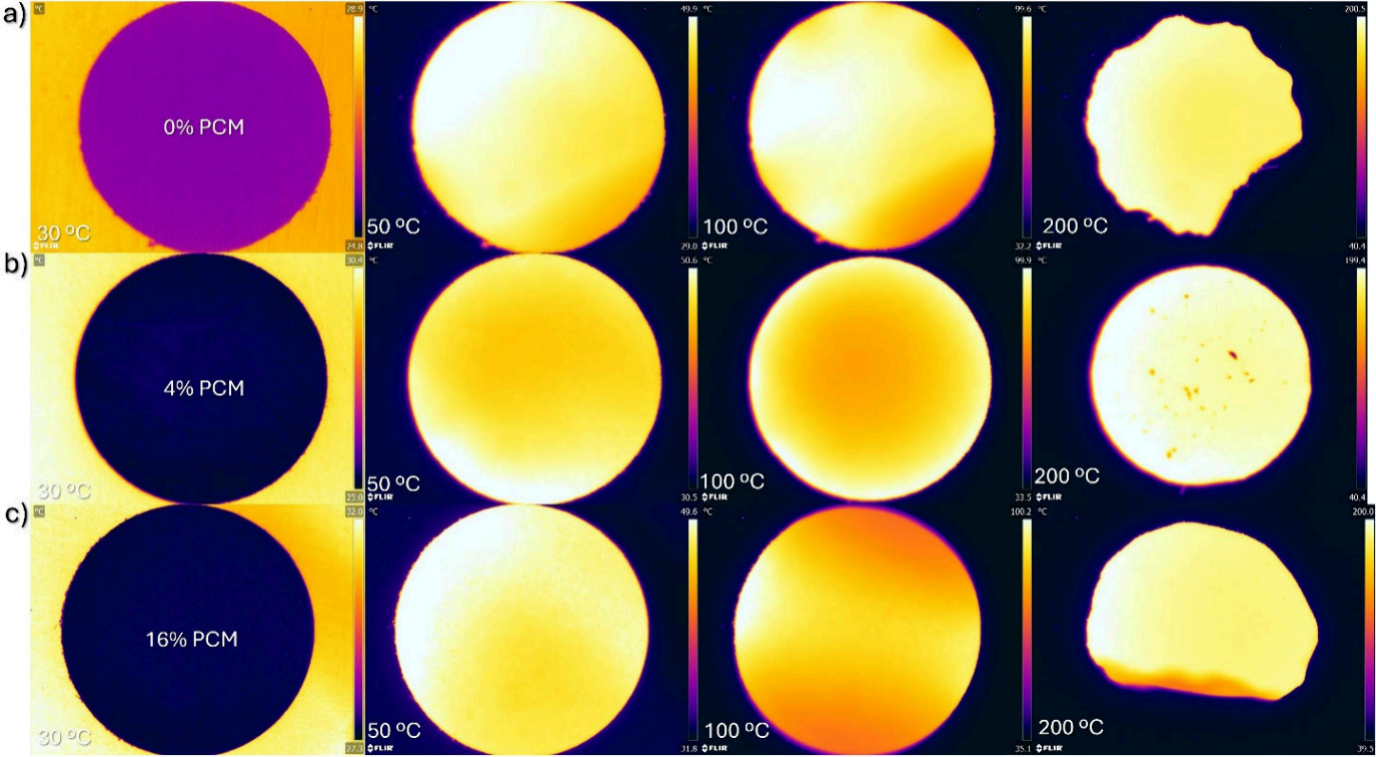
Optical images at different thermal temperatures, 30, 50, 100, and 200 °C, for composite separator membranes with (a) 0, (b) 4, and (c) 16 wt % microencapsulated PCM content, respectively. The diameter of the separators is 19 mm.

It is observed that composite membranes with PCM (Figure 5b,c) present uniform thermal distribution at different temperatures compared to neat PVDF-HFP (Figure 5a), demonstrating a homogeneous heat distribution on the surface of the separator, also correlated with a suitable distribution of the PCM microspheres, melting at ∼28.8 °C with low thermal shrinkage and consequently robust mechanical stability upon heating.^37^ Figure 5c shows that at 200 °C the membrane becomes curled in this freestanding state in the experiments, which will not occur on practical applications due to the mechanical constraint. In practical applications, this behavior preserves the separator’s integrity at high temperatures, which is crucial for reducing the risk of a short circuit.

### 3.3 Electrolyte Uptake and Electrochemical Characterization

Electrolyte uptake and electrochemical parameters, such as the ionic conductivity value and lithium transference number, will determine the functional characteristics of the separator membranes for battery applications. Electrolyte uptake for the developed membranes as a function of immersion time is shown in Figure 6a, showing a fast process, independently of the membrane, based on the strong interaction between the organic solvent present in the electrolyte and the polar functional groups of PVDF, especially when crystallized in the polar β-phase.^38,39^ Furthermore, a correlation is observed between electrolyte uptake and the porosity value, with the highest uptake value (∼600%) corresponding to the membranes with higher porosity and pore size (Figure 6a).

**Figure 6:**
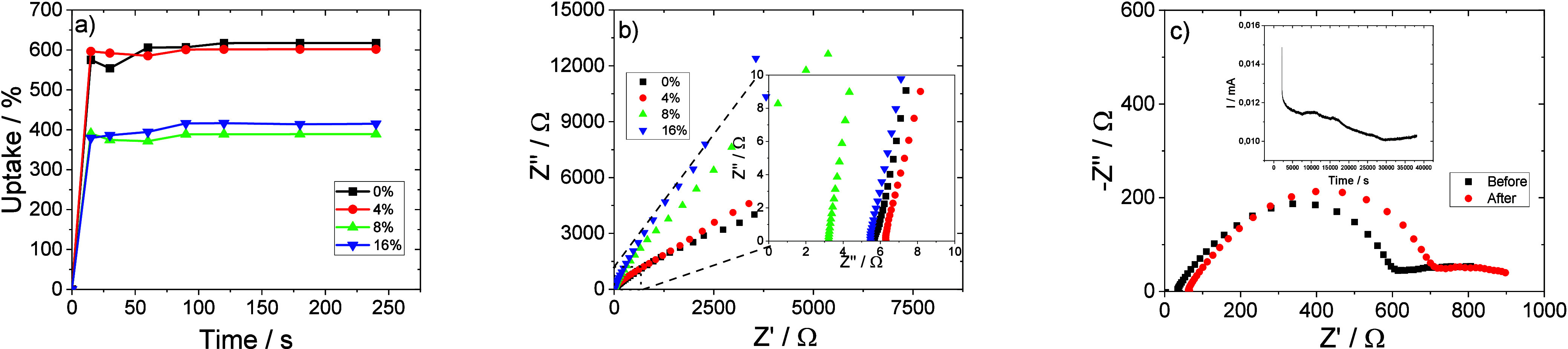
(a) Electrolyte uptake for the different membranes. (b) Nyquist plots at room temperature for all membranes. (c) DC polarization measurements for the PVDF-HFP membranes with 16 wt % PCM.

After the electrolyte uptake process, the ionic conductivity was obtained using the Nyquist plot at 25 °C as shown in Figure 6b. The Nyquist graph is characterized by an inclined straight line, which is related to the diffusion process of the polymer chains.^40^ From eq 4, the ionic conductivity was obtained and is presented in Table 2.

**Table 2:** Ionic conductivity (σi), tortuosity (τ), MacMullin number (N_M_) and lithium transference number (t_Li_^+^) values of the PVDF-HFP composite membranes

sample/(wt %)	σ_i_/(mS·cm^–1^) at RT ± 0.4	τ ± 2%	*N*_M_ ± 2%	*t*_Li_ ± 0.05
0	4.6	1.3	2.5	0.52
4	4.5	1.4	2.6	0.52
8	4.2	1.3	2.8	0.54
16	2.8	1.4	4.1	0.29

It is observed that the ionic conductivity value decreases with increasing PCM content, being related to the parameters of porosity and electrolyte uptake: the decrease in porosity and electrolyte uptake leads to a decrease in ionic conductivity. For the composite membranes, a maximum ionic conductivity of 4.5 mS·cm^–1^ is obtained for the membrane with 4 wt % PCM.

Table 2 also shows the tortuosity and MacMullin number (*N*_M_), which were determined by eqs 5 and 6, respectively. The tortuosity value of the membranes varies between 1.3 and 1.4, which is very close to the ideal value (τ = 1), which demonstrates that the conductivity path is uniform and parallel to the transport direction, related to the good pore connectivity.^40^ The values obtained for the MacMullin number (*N*_M_) range from 2.5 to 4.1, which is consistent with the uptake value of the electrolyte solution by the membranes; i.e., a low *N*_M_ number is obtained for a membrane with a high uptake value, as it is related to the ionic conductivity.

Using the Bruce–Evans approach from eq 7, Figure 6c displays the DC polarization measurements for the membrane containing 16 wt % PCM in order to determine the lithium transference number (*t*_Li^+^_). The corresponding values are shown in Table 2, and the obtained values are higher than those of commercial polypropylene (PP) separators (0.31)^41^ except for the membrane with 16 wt % PCM content, due to the lower electrolyte uptake and lower ionic conductivity value (2.8 mS·cm^–1^).

Considering the lithium transfer number and the ionic conductivity value obtained for the different membranes (Table 2), the cycling behavior was evaluated in cathode half-cells.

### 3.4 Cycling Behavior

Li/LFP Swagelok cells were assembled with the different composite membranes with PCM as separator membranes, and the corresponding charge–discharge behavior was evaluated at room temperature as represented in Figure 7. The charge–discharge profile for the 10th cycle for PVDF-HFP membranes with 16 wt % PCM content at different C-rates is shown in Figure 7a as a representative sample. For the other membranes, these profiles are similar.

**Figure 7:**
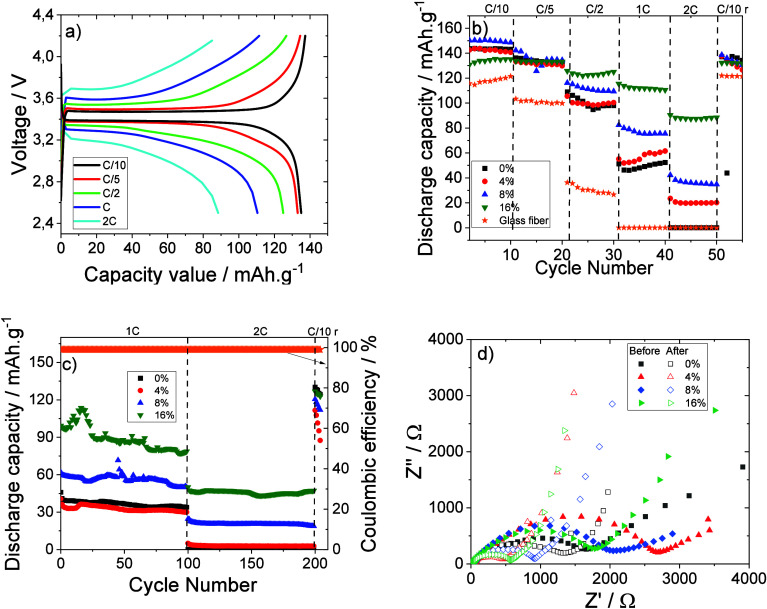
(a) Charge/discharge profile of the 10th cycle for membrane with 16 wt % PCM content at different C-rates. (b) Rate performance as a function of the number of cycles and (c) cycle life behavior after 100 cycles at C and 2C rates for all membranes. (d) Electrochemical impedance spectroscopy for all membranes prepared at room temperature before and after cycling process.

Independent of the scan rate and cycle number, a two-phase Fe^2+^/Fe^3+^ redox reaction between FePO_4_ and LiFePO_4_ is shown in Figure 7a. The characteristic flat voltage plateau is observed at 3.4 V^42^ up to the C/2 rate. At rates above C/2, an oblique line is instead detected that illustrates also a capacitive storage behavior.^43^

The rate performance for ten cycles for each rate, from C/10 to 2C rate, is shown in Figure 7b during the membranes’ discharge process and compared with a glass fiber separator, showing that the discharge capacity is stable as a function on the number of cycles, regardless of the C rate.

At 2C rate the obtained discharge capacities are 87, 36, 19, and 0 mAh·g^–1^ for the membranes with 16 wt %, 8 wt %, 4 wt %, and neat polymer membrane, respectively.

The discharge capacity value is excellent for all samples, improved with respect to the commercial glass fiber separator membrane, and there is not a direct correlation between the ionic conductivity value and the discharge capacity. The main reason for this behavior is that the higher PCM content does not improve the ionic conductivity value, but it guarantees the separator’s integrity and consequently improves the battery performance. This behavior is also related to the effect of PCM on membrane morphology as observed in Figure 2.

Taking into consideration the good rate capability of all membranes observed from Figure 7b, Figure 7c shows the cycling stability at C and 2C rates for 100 cycles for each cycle. The cycling behavior of the membranes follows the same pattern as observed in Figure 7b). For C rate (Figure 7c), good stability is observed throughout all cycles, except for the membrane with 16 wt % PCM content.

Regarding the 2C rate, the cycling behavior is practically constant for all membranes without capacity fading (Figure 7c). Furthermore, good reversibility and excellent Coulombic efficiency (∼95%) are also observed with a good discharge capacity value at the C/10 rate after 200 cycles.

Figure 7d shows the Nyquist plot before and after cycling for all membranes in order to evaluate the impact of the membranes on battery performance at room temperature.

In all Nyquist plots in Figure 7d, different regions are observed: the ohmic resistance at high frequencies, the global resistance in the medium frequency range, which corresponds to the charge transfer resistance at the solid–film interface, and the lithium resistance for ionic migration at the solid–electrolyte interface (SEI).^44^ Furthermore, in Figure 7d, at low frequencies an inclined line is observed representing the Warburg impedance correlated with Li^+^ diffusion.^44^ Before cycling (Figure 7d), the overall resistance for the neat PVDF-HFP, membrane with 16 wt % PCM content, membrane with 8 wt % PCM content, and membrane with 4 wt % PCM content is 1635, 1761, 2027, and 2701 Ω, respectively. After cycling, the overall resistance values decrease due to the formation of a stable solid–electrolyte interface (SEI layer), the value being 570 Ω for the membrane with 16 wt % PCM content. This behavior indicates improved interface stability due to the low resistance as a consequence of the good compatibility of the separator and electrodes, required to achieve excellent battery performance. The inclusion of the PCM within the membranes results in low thermal shrinkage and consequently mechanical stability of the separators upon heating, as confirmed with the higher discharge capacity in Figure 7b,c.

Considering that PCM microspheres improve battery performance through thermal regulation that results in low thermal shrinkage and that the presence of the PCM affects the morphology of the separators, the influence of temperature within the membrane morphology was studied. For that, Figure 8 shows the SEM surface images of the neat membrane and the membrane with 16 wt % PCM content before and after thermal treatment at 80 °C during 1 h, a temperature in which the battery security is compromised by thermal runaway processes. This morphology remains unchanged after cycling (Figure 8c).

**Figure 8:**
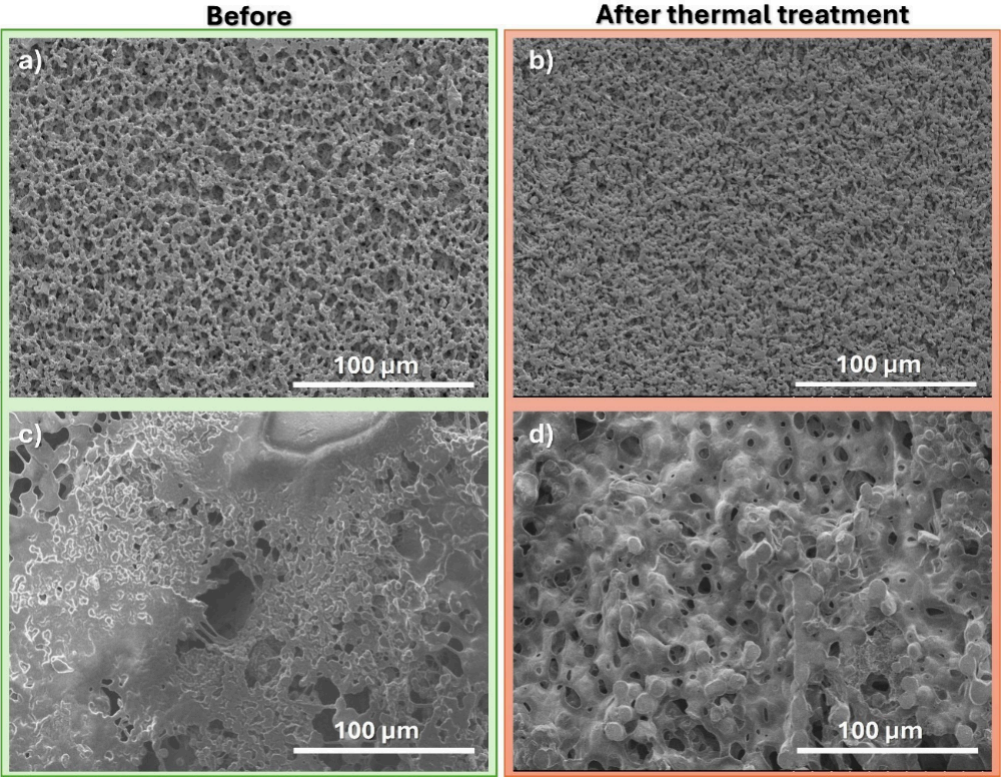
SEM surface images of (a, b) neat membrane and (c, d) membrane with 16 wt % PCM content, before and after thermal treatment at 80 °C.

For the neat membrane (Figure 8a,b), a slight effect on the morphology is observed while maintaining the porosity and pore size connectivity.

For the membrane with 16 wt % PCM content (Figure 8c,d), the morphology is completely different, where the microspheres increase the thermal mass and affect the microstructure by reducing the pore size. This behavior suggests that this composite membrane can act as a security and active battery component once the observed pore size reduction can decrease the lithium-ion percolation acting as a thermal shutdown. Furthermore, as observed from Figure 5, the integrity of the membranes is maintained even at high temperatures, preventing the short circuit of the battery and improving even more the battery safety.

Taking into account the excellent charge–discharge results shown in Figure 7, Table 3 compares the electrochemical characteristics of the membranes that best performed in our work with other PVDF-HFP membranes published in the literature for the LFP electrode.

**Table 3:** PVDF-HFP Membrane Composite Based Separator Membranes for LIB Applications

polymer matrix	fillers	electrolyte	porosity/uptake (%)	ionic condutivity (mS.cm^–1^)	discharge capacity (mAh.g^–1^)	ref
PVDF-HFP	alumina (Al_2_O_3_)	1 mol L^–1^ LiPF_6_ of EC, DMC, and ethyl methyl carbonate with a ratio of 30/15/35/20 + 2% VC)	—/∼373	1.3	93 at 2C	^45^
PVDF-HFP	alumina	1 M LiPF_6_ in 1:1:1 (wt %) EC–DMC–EMC	40/105	0.89	130 at 0.1C	^46^
PVDF-HFP	graphene oxide (GO)	1 M LiPF_6_ in EC/DMC solution = 1:1 (v:v)	86.8/342.4	0.42	—	^47^
PVDF-HFP	Li_1.7_Al_0.3_Ti_1.7_(PO_3_)_4_ (LATP)	1 M LiPF_6_ in EC:DMC = 1:1 vol %	—/140	0.94	135 at 2C	^48^
PVDF-HFP	silica (SiO_2_)	1.0 M LiPF_6_ in EC–diethyl carbonate (DEC)(1:1, v/v)	—/280	1.9	146 at 0.2C	^49^
PVDF-HFP	silica (SiO_2_)	0.5 M solution of LiTFSI salt dissolved into Pyr13TFSI	—/—	1.22	normalized discharge capacity is 83.3%	^50^
PVDF-HFP	silicon nitride (Si_3_N_4_) whiskers	1 M LiPF_6_ in EC/DMC solution = 1:1, v: v	75.2/—	0.884	103.9 at 5C	^51^

Table 3 demonstrates that the electrochemical results obtained in the present work are comparable to those published in the literature for other PVDF-HFP separator membranes. Whereas the membrane with PCM microspheres improves battery performance compared to the neat membrane in terms of discharge capacity value and lower capacity fading, PCM microspheres also improve thermal regulation and consequently the integrity of the separator through with low thermal shrinkage upon heating, which represents a main novelty and advantage of this work. In this work, we adequately addressed and solved the relevant issue of battery safety by thermal effects, leading to a promising strategy to obtain lithium-ion battery separators with improved safety without negatively affecting performance. Thus, this work also opens a route for the development of new and safer LIBs as the developed strategy can be applied in different battery types and with different form factors.

## 4 Conclusions

Thermal regulation was studied in battery separator membranes by including phase change materials (PCM) in poly­(vinylidene-*co*-hexafluoropropylene), PVDF-HFP, prepared by thermally induced phase separation (TIPS). The membranes were prepared with different contents of PCM microspheres up to 16 wt %. The content of PCM microspheres affects the porous microstructure with different average pore sizes and a degree of porosity ranging from 76 to 47%.

Furthermore, the inclusion of the PCM microspheres affects the content of the polar β phase, which decreases from 86 to 78% and the degree of crystallinity, which ranges between 22 and 13%, with increasing PCM content.

From optical images, it is demonstrated that the PCM content improves the thermal distribution of the membrane and, consequently, guarantees its integrity.

The uptake value, ionic conductivity, and lithium transfer number of the membranes are influenced by the PCM content where the membrane composed of 16 wt % PCM microspheres shows 400%, 2.8 mS·cm^–1^, and 0.28, respectively.

The electrochemical performance of the Li/C-LiFePO_4_ half-cells shows an excellent discharge capacity value at different C rates for all membranes, the cycling behavior being observed for the membrane with 16 wt % PCM microspheres of 87 mAh·g^–1^ after 200 cycles and 2C rate without capacity fade, where the PCM microspheres improve the thermal regulation of the separator in which presents low thermal shrinkage and, therefore, improved mechanical stability upon heating.

The excellent electrochemical performance of the developed membranes, which includes capacity retention and cyclability, indicated that they are suitable for lithium-ion batteries with thermal regulation properties, allowing them to prevent thermal runaway and, consequently, improve their safety.

## Supplementary Material



## Notes

Thermogravimetric analysis (TGA) plot of the composite separator membranes with 0 and 16 wt % PCM content (PDF)
