# Supplementary material for: Improving Thermal Regulation of Lithium-Ion Batteries by Poly(vinylidene fluoride-co-hexafluoropropylene) Composite Separator Membranes with Phase Change Materials
Source: ACS Appl Energy Mater. 2025 Jan 22;8(3):1847–56. doi: 10.1021/acsaem.4c03121 (PMC12821162; doi:10.1021/acsaem.4c03121)
Supplement: Supplementary file 1 [file ae4c03121_si_001.pdf]

Supporting information

Improving thermal regulation of lithium-ion batteries by poly(vinylidene fluoride-co-hexafluoropropylene) composite separator membranes with phase change materials

João P. Serra[1], Guilherme Antunes[1], Arkaitz Fidalgo-Marijuan[2,3], Manuel Salado[3,4], Renato Gonçalves[5], Weidong He[6], Senentxu Lanceros-Mendez[1,3,4], Carlos M. Costa[1,7*]

[1]Physics Centre of Minho and Porto Universities (CF-UM-UP) and Laboratory of Physics for Materials and Emergent Technologies, LapMET, University of Minho, 4710-057 Braga, Portugal.

[2]Department of Organic and Inorganic Chemistry, University of the Basque Country UPV/EHU, 48940 Leioa, Spain.

[3]BCMaterials, Basque Center for Materials, Applications and Nanostructures, UPV/EHU Science Park, 48940 Leioa, Spain.

[4]IKERBASQUE, Basque Foundation for Science, Bilbao, 48009, Spain.

[5]Centre of Chemistry, University of Minho, Braga, 4710-057, Portugal.

[6]National Key Laboratory of Science and Technology on Advanced Composites in Special Environments, and Center for Composite Materials and Structures, Harbin Institute of Technology, Harbin 150080, PR China

[7]Institute of Science and Innovation for Bio-Sustainability (IB-S), University of Minho, 4710-053, Braga, Portugal

*Corresponding author: C. M. Costa (cmscosta@fisica.uminho.pt)

TGA measurements were performed (Netzsch STA 449 F3 Jupiter equipment) under nitrogen atmosphere at a rate of 10 ºC.min^{-1} in the temperatures range from 25 to 700 ºC.

S-1: TGA plot of the composite separator membranes with 0 and 16 wt.% PCM content

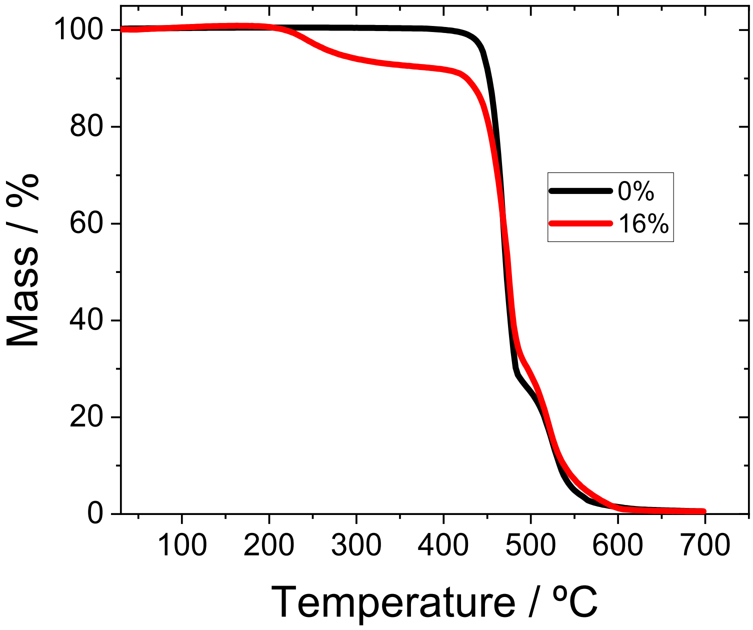

Figure S1 – TGA plot of the composite separator membranes with 0 and 16 wt.% PCM content.
